# PROTOCOL: Mass deworming for improving health and cognition of children in endemic helminth areas: a systematic review and individual participant data network meta‐analysis

**DOI:** 10.1002/CL2.212

**Published:** 2018-04-18

**Authors:** Vivian Welch, Elizabeth Ghogomu, Alomgir Hossain, Paul Arora, Simon Cousens, Michelle Gaffey, Alison Riddle, Rehana Salam, Peter Tugwell, Zulfiqar Bhutta, George A Wells, Advisory Group: Robert Black, Celia Holland, Deirdre Hollingsworth, Sue Horton, Sanjay Wijeseker

## Background

### The problem, condition or issue

Soil transmitted (or intestinal) helminths and schistosomes affect millions of children worldwide. There are four species of soil transmitted helminths: *Ascaris lumbricoides* (roundworm), *Necator americanus* and *Ancylostoma duodenale* (hookworms), and *Trichuris trichura* (whipworm). The five species of schistosomes which affect humans include: *Schistosoma (S.) mansoni*, *Schistosoma japonicum*, *Schistosoma mekongi*, *Schistosoma intercalatum* (which cause intestinal schistosomiasis) and *Schistosoma haematobium* (which causes urinary schistosomiasis).

Mass deworming is applied widely to reduce the consequences of helminth infection, and there have been numerous studies on the effects of deworming on growth, cognition and learning outcomes in children over the past several decades. Systematic reviews and meta‐analyses based on aggregate results of the effect of mass deworming on health and education outcomes are conflicting with some showing benefit (Hall, Hewitt et al. 2008; Croke, Hicks et al. 2016) and others not (Taylor‐Robinson, Maayan et al. 2015; Welch, Ghogomu et al. 2017). Debate has ensued about whether these conflicting results are due to the influence of variations in effect across individual‐level characteristics such as whether children are infected or not and intensity of infection (Hotez, Molyneux et al. 2007; Bundy, Kremer et al. 2009; Montresor, Addiss et al. 2015) as well as setting characteristics such as the sanitation environment and rapidity of reinfection (Campbell, Nery et al.).

### The intervention

Mass deworming for soil‐transmitted helminth infection and schistosomiasis is recommended one to four times per year in order to reduce worm burden in the updated World Health Organization in endemic areas, depending on prevalence of worm infection (WHO 2017). These updated WHO guidelines cite the Campbell and Cochrane systematic reviews on deworming which both concluded there were little to no effects of deworming on child welfare outcomes which included growth, anaemia and cognitive outcomes (Taylor‐Robinson, Maayan et al. 2015; Welch, Ghogomu et al. 2016). Mass deworming can be applied to school‐aged children or whole communities. Selective treatment of infected individuals is rarely done due to the high cost of screening for infection.

The drugs used include albendazole, mebendazole, levamisole, ivermectin and piperazine for soil‐transmitted helminth infection and praziquantel for schistosomiasis. These drugs are usually provided as pills, are inexpensive and can be administered by schoolteachers or parents. The drugs are considered to have few minor and transient side effects, such as gastrointestinal discomfort, headache, nausea, dizziness, oedema, myalgia and vomiting (WHO 2017).

Mass deworming is sometimes accompanied by iron, micronutrient or food supplementation in order to correct nutritional deficiencies that may have been caused by worm infections (Taylor, Jinabhai et al. 2001; Friis, Mwaniki et al. 2003; Nga, Winichagoon et al. 2009; de Gier, Campos Ponce et al. 2014; Rajagopal, Hotez et al. 2014). In addition water and sanitation measures may be implemented with mass deworming to reduce exposure and transmission of infections.

### How the intervention might work

Even with heavy infections, the nutritional requirements of intestinal worms relative to their human hosts are small. The harm to child welfare is expected to be caused by three factors: 1) malabsorption, 2) tissue damage and bleeding, and 3) loss of appetite (Crawley 2004). STH infections may cause malabsorption of nutrients in their hosts because of damage to the gastrointestinal surfaces. Hookworm infections are associated with anaemia, thought to be due to hookworm feeding on host tissue and to bleeding when they move from one site to another (Hall, Hewitt et al. 2008). Intestinal infections may also lead to reduced appetite which may negatively influence both anthropometric measures and attention in school.

**Figure 1:**
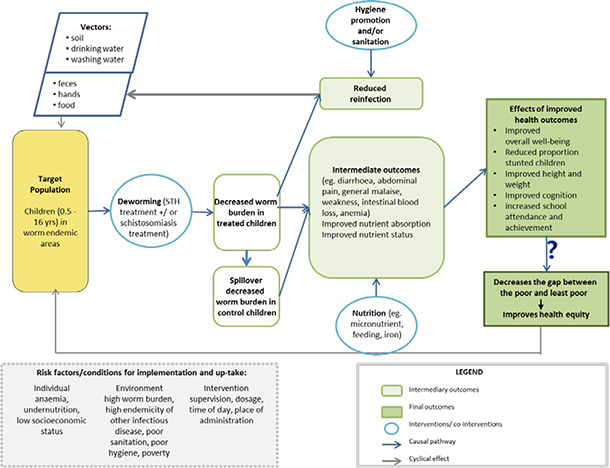
Logic model

Deworming drugs are over 90% effective at reducing the worm load in individuals, and are expected to reduce the prevalence of worm infection in the community as well as the intensity of infection in individuals (Figure 1). Reducing the prevalence and intensity of infection is expected to improve child nutritional status due to the mechanisms described above of reducing blood loss, reducing damage to gastrointestinal surfaces and improving appetite. Improved nutritional status and appetite are expected to improve attention in school and cognitive outcomes. Some have argued that deworming alone is insufficient to improve child welfare outcomes since the nutritional deficiencies caused by infections must be corrected with food and/or micronutrients (Hall, Hewitt et al. 2008).

Many potential effect modifiers have been described in the literature. Younger children may have a greater impact of deworming since they are smaller in size and the impact of infections may be greater on them (Hall, Hewitt et al. 2008). Girls may benefit less from deworming if they have lower school attendance (thus, not receiving deworming given at school) and if there is preferential distribution of food or other resources at home which could influence child welfare. Children who are stunted for age at three years of age may not be able to benefit as much in terms of growth. Conversely, children who are underweight may benefit more from deworming than those of normal weight (Hall, Hewitt et al. 2008). It is expected that benefits of deworming would only accrue to those who are infected, and those with heavier infection intensity (Hall, Hewitt et al. 2008). Low socioeconomic status is expected to be correlated with other features that expose children to repeat intestinal infections, including those that cause diarrhea, and thus children with lower socioeconomic status may not achieve as much benefit as less poor children.

Reinfection is expected to depend on the prevalence and intensity of infection as well as environmental factors such as the water and sanitation environment and hygiene practices in the community.

### Why it is important to do the review

A recent Campbell systematic review and network meta‐analysis by members of our team (VW, PT, GAW, EG, ZB), with 47 randomized trials and >1 million children, found little to no overall effect on growth, attention and school attendance (Welch, Ghogomu et al. 2016). With network meta‐analysis, we were able to explore the size of effect with different types and frequency of drugs and their combination with food or micronutrients; none of which contributed to larger effects. Our review also did not find larger effects in subgroups of children at the aggregate level across characteristics such as age, baseline nutritional status, prevalence or intensity of infection that have been postulated to be important (Welch, Ghogomu et al. 2016). These analyses were conducted at the study level, rather than using data for each individual child, which limits the power to detect effect modification by individual participant characteristics and also may be subject to ecological bias. This review was therefore unable to identify whether mass deworming was more effective for children with certain characteristics. There was substantial unexplained heterogeneity between studies, with some studies finding larger effects than others, and no single individual‐level, setting‐level or methodology characteristic explaining this variation. Thus, we concluded that our analysis of effect modifiers was limited by the aggregate level data.

Our previous review was conducted using network meta‐analysis (NMA), which allowed the comparison of treatments which had not been directly compared in head‐to‐head trials. NMA also allowed for the assessment of the role of multi‐component interventions (such as deworming combined with other parasite control interventions, food or micronutrients). Because there are several drugs used for mass deworming, this allowed the assessment of heterogeneity related to the type of drug, frequency and use of concomitant interventions.

Individual participant data (IPD) meta‐analysis has been called the “gold standard” in meta‐analyses for exploring individual level characteristics and their association with effects (Stewart 1995). Advantages of IPD meta‐analysis include improving data quality, enabling standardization of outcomes, clarifying risk of bias, and increasing the power to assess the interaction of participant characteristics with effect size (Stewart, Clarke et al. 2015). Furthermore, IPD analysis can explore the size and direction of differences in effect, thus assessing whether there is a greater benefit for some participants (Early Breast Cancer Trialists’ Collaborative Group 1990). Another advantage of IPD is that they usually require an international collaborative effort, involving trial authors, which may help to identify more relevant trials, and also contribute to an agreed analysis plan and shared understanding of the results.

While failure to obtain some datasets may lead to selection bias if there are systematic reasons why some studies do not provide full data, methods have been developed to combine individual participant data with aggregate data (when IPD is not available for some studies) in network meta‐analysis (Sutton, Kendrick et al. 2008; Donegan, Williamson et al. 2012).

We decided in collaboration with several authors of primary trials that there would be value in conducting an IPD meta‐analysis to explore the question of whether mass deworming is more effective for subgroups of children defined by characteristics such as infection intensity or status, age or nutritional status. This understanding could help to develop targeted strategies to reach these children better with deworming and guide policy regarding deworming.

## Objectives

The primary objective is to use individual participant data network meta‐analysis to explore whether the effects of different types and frequency of deworming drugs as well as their combination with food or micronutrients on anaemia, cognition and growth vary with child‐level and environment‐level characteristics (see Table 1), specifically: intensity of infection (as assessed by egg count), infection status (including species of worm), age, nutritional status, socioeconomic status and sanitation environment.

**Table 1:** Potential effect modifiers at child‐level and environment level

Child‐level	Environment
Age	Population level prevalence
Sex	Population level intensity
Nutritional Status	Water and sanitation environment
Infection status	
Socioeconomic status	
Intensity of infection (including type of worm and duration of infection)	

^*^Environment‐level factors will not be entered into the same model as individual‐level modifiers because these factors are likely multi‐collinear. Instead these factors will be explored with sensitivity analysis.

## Methodology

We report this protocol according to the preferred reporting items for systematic reviews and meta‐analyses for protocols (PRISMA‐P) (Moher, Shamseer et al. 2015). Results of the review will be reported using the Preferred Reporting items for Systematic Reviews and Meta‐analyses of individual patient data (PRISMA‐IPD) Statement (Stewart, Clarke et al. 2015).

### Criteria for including and excluding studies

We will include studies which meet the following eligibility criteria:

#### Types of study designs

We will include randomized and quasi‐randomized trials. For the purpose of determining whether specific individual‐level and environment‐level characteristics are associated with greater effects of deworming, there is sufficient evidence from over 70 randomized trials with over 100,000 children to include only randomized and quasi‐randomized trials. We will include studies reported in abstract form at a conference as well as unpublished studies. We will seek full datasets from all studies and carry out the same methods for data checking and quality for all studies.

#### Types of participants

Children aged six months up to 16 years. We will exclude studies with less than 100 participants because of the time and effort required for each dataset and the information gained from smaller studies will be small compared to larger datasets. We will not exclude studies on the basis of attrition rate from the study.

#### Types of interventions

Mass deworming using any drugs for soil transmitted helminths or schistosomes with or without co‐interventions such as food, micronutrients, iron or hygiene interventions. Eligible drugs include (but are not limited to) albendazole, praziquantel, levamisole, ivermectin, diethyl carbamazine, pyrantel, piperazine, metrifonate, hycanthone and tetramisole.

We will include studies with combined approaches to parasite elimination such as albendazole and praziquantel. Also, because deworming may be used in combination with iron, food or hygiene promotion, we will include studies with multiple component interventions.

Studies will be included with placebo, control, or other active interventions (e.g. vitamin A, iron, hygiene promotion) as comparators.

As network meta‐analysis depends on the assumption of transitivity (that participants could be randomized to any one of the treatments) (Salanti 2012), we will consider two evidence networks of jointly randomizeable interventions of drugs given for two indications. First, we will assess the evidence network of interventions given for soil‐transmitted helminths which includes different frequencies of albendazole, mebendazole, levamisole, pyrantel, piperazine, ivermectin and tetramisole with or without micronutrients or food. These are considered jointly randomizable because they are given for the same indication, and many have been compared in multi‐arm trials (Salanti 2012). Secondly, we will consider the evidence network of interventions given for schistosomiasis (praziquantel, metrifonate, hycanthone) with or without micronutrients or food.

#### Types of outcome measures

The primary health outcomes are change from baseline in: weight (kg), height (cm), serum ferritin, cognition and hemoglobin (g/L). We will include studies which measure weight, hemoglobin, serum ferritin, cognition or height. Cognition may be measured using scales that measure development (e.g. Raven's matrices) or tests that assess attention using digit recall.

We will not exclude on the basis of reported outcomes since some measured outcomes may not be reported in trial reports or abstracts.

We will not assess distal outcomes for this review because there is too little data available so an IPD NMA would not add to the literature.

We will use the available data on age and sex to calculate height for age, weight for age and weight for height for children <5 years using the 2006 child growth standards (using WHO software Anthro version 3.2.2) and body mass index (BMI) for age for children aged five or older using the WHO Reference 2007 (using WHO AnthroPlus software).

Effects on infection intensity and status will be assessed as secondary outcomes. Adverse effects of deworming were assessed in prior systematic reviews as minor and uncommon and the results are not contested, thus we will not assess adverse effects in this review.

Since the primary objective of this systematic review is to assess effect modification, particularly as it relates to infection status and intensity, we will exclude studies that do not measure baseline infection prevalence of at least one of the soil‐transmitted helminths or schistosomes.

#### Duration of follow‐up

For weight and height, we will include data from studies >4 months in duration because we consider this as a minimum duration to observe differences in growth. However, for hemoglobin and ferritin status, changes may occur sooner, so study duration will not be used as an exclusion criterion. While infection status and infection intensity are affected much sooner than this, these are not primary outcomes of interest since there is no question that deworming drugs reduce infection load. We will assess infection intensity and status at baseline as indicators of the force of infection in the population. We will collect data at each available time‐point and explore study duration as a covariate in a meta‐regression model, if possible.

#### Types of settings

The settings will include any area where soil‐transmitted helminths or schistosomes are endemic.

### Search strategy

We will use the same search strategy as used for a previous Campbell review by members of our team (Welch, Ghogomu et al. 2016). This search was last run on January 14, 2016. See search strategy in Appendix. We will search in the following databases: MEDLINE, CINAHL, LILACS, EMBASE, the Cochrane Library, Econlit, Internet Documents in Economics Access Service (IDEAS), Public Affairs Information Service (PAIS), Social Services Abstracts, Global Health CABI and CAB Abstracts.

Grey literature databases will include thesis dissertations and the System for Information on Grey Literature in Europe (SIGLE)‐ends in 2005). We will search websites of relevant organizations such as the World Bank, World Food Program and International Food Policy Research Institute, as per the prior Campbell review (Welch, Ghogomu et al. 2016).

We will also contact authors of studies and members of our advisory board for any unpublished studies or grey literature reporting eligible studies. We will check reference lists of relevant studies and reviews.

Titles and abstracts will be screened in duplicate by two reviewers. We will pilot‐test the screening criteria at both title and abstract screening stage and full text stage. We will use the PRISMA flow diagram to report eligibility of studies. We will retrieve full text of all studies which pass this first level screening. The full text review will also be done in duplicate by two reviewers, and agreement will be reached by consensus. Disagreements will be resolved by consultation with a third reviewer. No language limits will be applied. The research team has expertise in English, French and Spanish, and translation will be sought if studies are found in other languages.

### Description of methods used in primary research

Randomized controlled trials of deworming include two‐arm trials as well as factorial trials, with children allocated either individually or by cluster‐randomization (e.g. by village or school).

### Details of study coding categories

Details of the populations, interventions, comparators, outcomes and study design will be extracted in duplicate by two reviewers, using a pre‐tested form, designed for a previous Campbell review on deworming for children (Welch, Ghogomu et al. 2016). This extraction includes details about the context, setting and environment, as well as sociodemographic details, and details about the frequency, delivery method and dose of interventions.

Two independent reviewers will appraise each study with the Cochrane risk of bias tool which assesses selection bias, performance bias, detection bias, attrition bias and reporting bias (Cochrane Handbook) (Higgins, Altman et al. 2011). Disagreements will be resolved by discussion or consultation with a third reviewer.

We will appraise the GRADE certainty for each outcome for each comparison by two independent reviewers, using the GRADE approach for network meta‐analysis (Puhan, Schünemann et al. 2014). GRADE certainty (quality) “reflects our confidence that the estimates of the effect are correct. In the context of recommendations, quality reflects our confidence that the effect estimates are adequate to support a particular recommendation. “Quality as used in GRADE means more than risk of bias and so may also be compromised by imprecision, inconsistency, indirectness of study results, and publication bias” (Balshem, Helfand et al. 2011). The two reviewers will discuss ratings and reach consensus. Disagreements will be resolved by consulting a third reviewer.

We will develop a summary of findings table for each main comparison to show the effects for the outcomes of weight, height and haemoglobin, along with the quality of evidence (using GRADE certainty).

### Statistical procedures and conventions

Data will be prepared into a flat spreadsheet with the same fields for every study. We will consider the missing values for each variable as missing at random (MAR).

We will use multiple imputation to impute the missing values for baseline and outcome variables using Proc MI in SAS/STAT(SAS Institute Inc., Cary, NC, USA).

Descriptive characteristics of each study will be presented, with details on the child characteristics, environment, worm species, prevalence, and intensity of infection, geographic location, interventions, comparator and outcomes and risk of bias assessment.

We expect to have data from individually randomized trials and cluster‐randomized trials. We will account for clusters (such as villages, schools or households) as nested within each study.

We will analyse IPD datasets to check for comparability with the primary published papers. We will calculate the standardized difference between the published data and the IPD received from authors for baseline characteristics and baseline outcome assessment. For endline, we will replicate the effect measures reported in study publications and calculate the standardized difference between the IPD received and the study report (Austin 2009). Any differences larger than a standardized difference of 0.2 (chosen because it represents a small effect size) will be discussed with authors to attempt to resolve differences. If the reason for differences cannot be explained, the data will not be used or will only be used in sensitivity analyses.

As with our previous Campbell review, we will use a two‐step process to meta‐analysis. We will first conduct pairwise analyses for each comparison of interest by entering all IPD data into a multilevel model, with each study as one cluster. We expect considerable heterogeneity between studies for each outcome based on our Campbell review; therefore, we will use a random effects model. We will assess mean differences in change from baseline for weight (kg), height (cm), serum ferritin (mcg/l), and haemoglobin (g/l).

For cognition, we will analyse measures of motor and cognitive development separately. We will analyse measures of attention separately from developmental outcomes. We expect that cognition will be measured using different scales. We will not combine different measures of cognition.

If IPD are not available for all trials (as we expect), we will compare study characteristics and the effect sizes from aggregate data of studies which do not provide IPD to the studies which do provide IPD.

We will account for clustering as above by nesting clusters within studies. We decided on a set of pre‐defined covariates with advice from our advisory board and co‐authors. We will account for the covariates of sex, age, infection intensity for each type of agent, socioeconomic status, maternal education and baseline nutritional status in the model. We will assess heterogeneity using visual inspection of forest plots for pairwise analyses as well as statistical tests of heterogeneity (I^2^).

**Figure 2:**
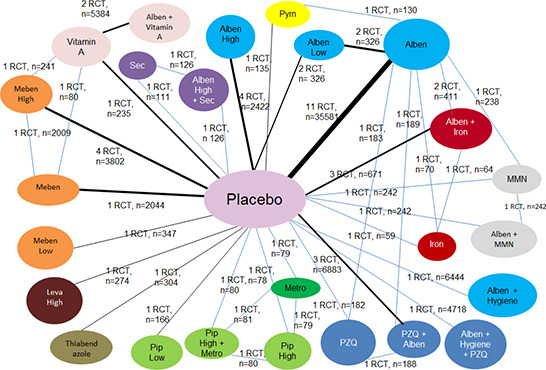
Evidence Network for weight (n=29 randomized trials, 61,857 participants)

We will conduct network meta‐analysis with IPD, using a frequentist approach for random‐effects network meta‐analysis. The covariates were identified by the Study Advisory Group, namely: age, sex, baseline nutritional status (weight and height), haemoglobin, and infection intensity. The effect estimate chosen is the mean difference for the continuous outcome of interest. The response variables (weight, height and haemoglobin) follow the normal distribution, and the generalized linear model will be used to fit data to determine the parameter estimate.. Random effect GLMM will be conducted with two random effects considered in the model: random effect ‘trial’ accounts for the response variables of patients within a given trial being correlated; and random effect ‘trial*treatment’ accounts for the correlation of responses between any two patients from the same treatment arm within a given trial, subject to sufficient data available. We expect to have a connected network of trials to allow direct and indirect comparisons based on our Campbell review and network meta‐analysis (see Figure 2 for evidence network diagram for weight) (Welch, Ghogomu et al. 2016). We will use the GLIMMIX procedure in SAS/STAT (SAS Institute Inc., Cary, NC, USA) for the GLMM network meta‐analysis, considering models that account for multi‐arm trials and adjust for the covariates identified. Results will be summarized as point estimates with 95% confidence intervals.

### Assessment of clinical and methodological heterogeneity within treatment comparisons

We will compare heterogeneity of participant characteristics and trial methodology in tables. Within GLM, the explanatory model will include covariates at the study level and patient characteristics (anaemia, nutritional status, infection intensity, age, sex). We will construct forest plots for treatment comparisons (adjusted using the same covariates as the base case model) and assess heterogeneity by visual inspection and homogeneity statistics.

Any environment level or participant‐level covariates that are statistically significant will be analyzed using subgroup analyses.

### Assessment of transitivity across treatment comparisons

Transitivity cannot be assessed statistically. With IPD, we have more opportunity to account for and model heterogeneity. As proposed by Salanti 2012, we will use IPD to assess the distribution of the child‐level effect modifiers from Table 1 in each comparison to assess the plausibility of the transitivity assumption (Salanti 2012). As above, transitivity is considered plausible since the treatments in each model (STH and schistosomiasis, respectively) are provided for the same indication and many of the treatments and their cointerventions have been included in multi‐arm trials (Welch, Ghogomu et al. 2016).

### Assessment of statistical heterogeneity

For this IPD NMA, we assume equal variances across comparisons within network. This assumption will be tested using the Levene test (Gastwirth, Gel et al. 2009).

### Assessment of statistical inconsistency

Inconsistency in an NMA is defined as a disagreement between the direct estimates (from direct comparisons of treatments) and indirect estimates (which are derived from the network comparisons). We will assess underlying assumptions including consistency using back‐calculation technique (Dias, Welton et al. 2010; Wilson, Tanner‐Smith et al. 2016) between indirect and direct evidence and will use model diagnostics, including diagnostic plots (e.g. residual plots), to assess model convergence.

We will use node‐splitting (Dias, Welton et al. 2010; Dias, Welton et al. 2013) to assess consistency of the NMA. These statistical tests will be interpreted with caution since they are underpowered and their power varies with the heterogeneity in the pairwise comparisons.

### Publication bias

A funnel plot will be plotted for comparisons and outcomes with >10 studies. We will use Egger's test for asymmetry and visual inspection to assess the presence of publication bias and/or selective reporting.

We do not plan to rank interventions because there is controversy as to the utility of ranking.

**Subgroup analyses:** Provided sufficient data is available to inform the evidence network, meta‐regression and/or sub‐group analyses will be conducted to assess effects across both child‐level as well as environment‐level characteristics. We will compare the results of models with subgroup analyses by assessing the size of quantitative or qualitative differences in effects, the statistical significance of tests for interactions, assessing between‐study variance and assessing the goodness of fit of the models using the likelihood ratio.

The following child and environment level effect modifiers will be assessed:

***Child level:***

- Individual‐level intensity of infection with *Ascaris, trichuris* and hookworm (across four levels of none, light, moderate and heavy, using the WHO cutoffs for each helminth, available at: http://apps.who.int/iris/bitstream/10665/44671/1/9789241548267_eng.pdf)
- Stunting (HAZ>‐2, HAZ <‐2 to ‐3, HAZ <‐3),
- Undernutrition (defined by WAZ cutoffs for children <5 years of age (http://apps.who.int/iris/bitstream/10665/44129/1/9789241598163_eng.pdf?ua=1) and by BMI cutoffs for children aged 5 years or older available at http://www.who.int/growthref/who2007_bmi_for_age/en/),
- Anaemia (using WHO cutoffs by age and altitude of non‐anaemic, mild, moderate and severe, http://www.who.int/vmnis/indicators/haemoglobin.pdf)
- Age (<5 years, and ≥5 years of age
- Sex (male/female)
- Socioeconomic status: Socioeconomic status is measured in different ways in studies (e.g. questionnaires, asset indices, quintiles). We will assess whether the measurement of socioeconomic status can be compared across study settings and time. If so, we will conduct a sensitivity analysis with children in the poorest tertile.

Before conducting subgroup analyses, we will assess the distribution of each variable. If there are insufficient children in some categories, the levels may be combined.

If possible, we will also assess socioeconomic status of household or parents and maternal education as effect modifiers.

***Environment level***:

- study level sanitation and hygiene environment, as reported by studies will be assessed to consider whether environments can be classified according to consistent system
- study‐level prevalence (using WHO cut‐offs for each worm‐type, as above)
- study‐level intensity of infection (using WHO cut‐offs for each worm‐type, as above)

As noted in Table 1, environment level characteristics will not be entered into the model. They will be assessed by sensitivity analyses

We expect poor reporting on these details in the articles based on our prior Campbell review, but some studies may have collected information on this at the study level that were not reported in the paper publications. We will also assess whether there is sufficient data on the geographic location and date of the studies to assess study‐level prevalence generated by the Global Atlas of Helminth Infections (GAHI).

**Sensitivity analyses**

Provided sufficient data is available to inform the evidence network, we will conduct sensitivity analyses to assess robustness of results when restricted to studies at low risk of bias for sequence generation, allocation concealment and blinding of participants. We will assess whether results are robust to excluding imputed data (i.e. complete case analysis). We will assess sensitivity to restricting to studies published in 2008 or later (last 10 years).

Data will be housed at a secure data warehouse at the Bruyère Research Institute, following the personal health information act. Data will be transferred to SAS as a common platform for all studies, using a common data dictionary. VW will check IPD data for consistency immediately upon receiving datasets. For example, we will check for outlier individuals (e.g. with ages outside of eligibility criteria, duplicate participant IDs, unrealistic date ranges). We will compare the IPD from authors with the aggregate data reported in the articles. Any missing or unusual data will be flagged for discussion with the trial author or statistician by VW. We will ask for clarification from the authors to establish reasons for the errors, and correct them if possible. Any requests for authors will be discussed when the data is provided, such as clarification of trial risk of bias, conduct or eligibility criteria. We will also run the same statistical analysis as the authors to check for consistency with the published paper (Stewart, Clarke et al. 2015).

We will request statements of ethics approval from each study and we will not include data from studies that did not receive ethics approval. We will request that all data be transferred without any identifiers.

### Treatment of qualitative research

We do not plan to include qualitative research.

### Review authors

**Lead review author:** The lead author is the person who develops and co‐ordinates the review team, discusses and assigns roles for individual members of the review team, liaises with the editorial base and takes responsibility for the on‐going updates of the review.

**Table:** 

Name:	Vivian Welch, PhD
Title:	Clinical Investigator, Director, Methods Centre
Affiliation:	Bruyére Research Institute
Address:	85 Primrose Ave
City, State, Province or County:	Ottawa, Ontario
Post code:	K1R 6M1
Country:	Canada
Phone:	(613) 562‐6262 ext 2904
Email:	vwelch@campbellcollaboration.org
**Co‐authors**:	
Name:	Elizabeth Ghogomu
Title:	Research Associate
Affiliation:	Bruyére Research Institute
Address:	85 Primrose Ave
City, State, Province or County:	Ottawa, Ontario
Post code:	K1R 6M1
Country:	Canada
Phone:	(613) 562 6262 ext 2962
Email:	etanjongghogomu@bruyere.org
	
Name:	Alomgir Hossain
Title:	Assistant Professor, School of Epidemiology and Public Health
Affiliation:	Cardiovascular Research Methods Centre, University of Ottawa Heart Institute
Address:	40 Ruskin St
City, State, Province or County:	Ottawa, Ontario
Post code:	K1Y 4W7
Country:	Canada
Phone:	(613) 696 7000 ext 10633
Email:	ahossain@ottawaheart.ca
	
Name:	Paul Arora
Title:	Assistant Professor
Affiliation:	Dalla Lana School of Public Health, University of Toronto
Address:	155 College St
City, State, Province or County:	Toronto, Ontario
Post code:	M5T 3M7
Country:	Canada
Phone:	(647) 407 4867
Email:	paul.arora@utoronto.ca
	
Name:	Simon Cousens
Title:	Professor of Epidemiology and Medical Statistics
Affiliation:	London School of Hygiene and Tropical Medicine (LSHTM)
Address:	Keppel St
City, State, Province or County:	London
Post code:	WC1E 7HT
Country:	United Kingdom
Phone:	+44 (20) 7927 2422
Email:	Simon.Cousens@lshtm.ac.uk
Name:	Michelle Gaffey
Title:	Senior Research Manager
Affiliation:	Hospital for Sick Children, University of Toronto
Address:	555 University Avenue
City, State, Province or County:	Toronto, Ontario
Post code:	M5G 1X8
Country:	Canada
Phone:	
Email:	michelle.gaffey@sickkids.ca
	
Name:	Alison Riddle
Title:	Health and Gender Equality Advisor
Affiliation:	University of Ottawa
Address:	
City, State, Province or County:	Ottawa, Ontario
Post code:	
Country:	Canada
Phone:	
Email:	alison.riddle@gmail.com
	
Name:	Rehana Salam
Title:	Senior Instructor, Research
Affiliation:	Aga Khan University
Address:	
City, State, Province or County:	Karachi
Post code:	
Country:	Pakistan
Phone:	
Email:	rehana.salam@aku.edu
	
Name:	Peter Tugwell
Title:	Professor of Medicine, and Epidemiology & Community Medicine
Affiliation:	University of Ottawa
Address:	
City, State, Province or County:	Ottawa, Ontario
Post code:	
Country:	Canada
Phone:	
Email:	tugwellb@uottawa.ca
	
Name:	Zulfiqar Bhutta
Title:	Co‐Director, Centre for Global Child Health
Affiliation:	Hospital for Sick Children, University of Toronto
Address:	555 University Avenue
City, State, Province or County:	Toronto, Ontario
Post code:	M5G 1X8
Country:	Canada
Phone:	(416) 813 7654 ext 328532
Email:	zulfiqar.bhutta@SickKids.ca
	
Name:	George A Wells
Title:	Professor, School of Epidemiology and Public Health
Affiliation:	University of Ottawa Heart Institute
Address:	40 Ruskin St
City, State, Province or County:	Ottawa, Ontario
Post code:	K1Y 4W7
Country:	Canada
Phone:	613 696‐7000, X18640
Email:	gawells@ottawaheart.ca
	
Name:	Sue Horton
Title:	
Affiliation:	University of Waterloo
Address:	
City, State, Province or County:	Waterloo, Ontario
Post code:	
Country:	Canada
Phone:	
Email:	sehorton@uwaterloo.ca
	
Name:	Deirdre Hollingsworth
Title:	
Affiliation:	NTD Modelling Consortium
Address:	
City, State, Province or County:	
Post code:	
Country:	
Phone:	
Email:	Deirdre.Hollingsworth@warwick.ac.uk
	
Name:	Celia Holland
Title:	
Affiliation:	Trinity College Dublin
Address:	
City, State, Province or County:	
Post code:	
Country:	
Phone:	
Email:	CHOLLAND@tcd.ie
	
Name:	Sanjay Wijesekera
Title:	
Affiliation:	UNICEF
Address:	
City, State, Province or County:	
Post code:	
Country:	
Phone:	
Email:	swijesekera@unicef.org
	
Name:	Robert Black
Title:	
Affiliation:	Johns Hopkins University, Bloomberg School of Public Health
Address:	
City, State, Province or County:	
Post code:	
Country:	
Phone:	
Email:	rblack1@jhu.edu

### Roles and responsibilities

- Content: Michelle Gaffey, Zulfiqar Bhutta, Robert Black, Celia Holland, Deidre Hollingsworth, Sue Horton, Sanjay Wijesekera
- Systematic review methods: Vivian Welch, Elizabeth Ghogomu, Paul Arora, Alison Riddle, Rehana Salam, Peter Tugwell
- Statistical analysis: Alomgir Hossain, Simon Cousens, George A Wells
- Information retrieval: Jessie McGowan (search was designed for prior review by JM)

### Sources of support

This review is funded by the Bill and Melinda Gates Foundation (Funding reference number: OPP1140742).

### Declarations of interest

Michelle Gaffey, Robert Black, Deidre Hollingsworth, Sue Horton, Paul Arora, Alison Riddle, Rehana Salam, Simon Cousens have no conflict of interest, financial or otherwise that may influence judgments made in this review.

Celia Holland is a co‐author and principal investigator on a randomized trial of deworming: Kirwan et al 2009 (Kirwan, P., Asaolu, S. O., Molloy, S. F., Abiona, T. C., Jackson, A. L., & Holland, C. V. (2009). Patterns of soil‐transmitted helminth infection and impact of four‐monthly albendazole treatments in preschool children from semi‐urban communities in Nigeria: a double‐blind placebo‐controlled randomised trial. BMC infectious diseases, 9(1), 20.)

Vivian Welch, Elizabeth Ghogomu, Alomgir Hossain, Jessie McGowan, Zulfi Bhutta, Peter Tugwell and George Wells are authors of the Campbell systematic review and network meta‐analysis of mass deworming for children (Welch, Ghogomu et al. 2016).

Vivian Welch is Editor in Chief of the Campbell Collaboration.

### Preliminary timeframe

Approximate date for submission of the systematic review: June 2018.

**Table:** 

	Oct 2017	Nov	Dec	Jan 2018	Feb	Mar	April	May	Jun	Jul	Aug
Protocol submission	X										
Searching and screening	X	X									
Data extraction		X	X								
Synthesis			X	X	X	X	X				
Interpretation and write up					X	X	X	X	X		
Publication									X	X	X

Please note this should be no longer than two years after protocol approval. If the review is not submitted by then, the review area may be opened up for other authors.

### Plans for updating the review

We will consider updating or taking part in an update should resources be made available.

### AUTHOR DECLARATION

#### Authors’ responsibilities

By completing this form, you accept responsibility for preparing, maintaining and updating the review in accordance with Campbell Collaboration policy. The Campbell Collaboration will provide as much support as possible to assist with the preparation of the review.

A draft review must be submitted to the relevant Coordinating Group within two years of protocol publication. If drafts are not submitted before the agreed deadlines, or if we are unable to contact you for an extended period, the relevant Coordinating Group has the right to de‐register the title or transfer the title to alternative authors. The Coordinating Group also has the right to de‐register or transfer the title if it does not meet the standards of the Coordinating Group and/or the Campbell Collaboration.

You accept responsibility for maintaining the review in light of new evidence, comments and criticisms, and other developments, and updating the review at least once every five years, or, if requested, transferring responsibility for maintaining the review to others as agreed with the Coordinating Group.

#### Publication in the Campbell Library

The support of the Coordinating Group in preparing your review is conditional upon your agreement to publish the protocol, finished review, and subsequent updates in the Campbell Library. The Campbell Collaboration places no restrictions on publication of the findings of a Campbell systematic review in a more abbreviated form as a journal article either before or after the publication of the monograph version in Campbell Systematic Reviews. Some journals, however, have restrictions that preclude publication of findings that have been, or will be, reported elsewhere and authors considering publication in such a journal should be aware of possible conflict with publication of the monograph version in Campbell Systematic Reviews. Publication in a journal after publication or in press status in Campbell Systematic Reviews should acknowledge the Campbell version and include a citation to it. Note that systematic reviews published in Campbell Systematic Reviews and co‐registered with the Cochrane Collaboration may have additional requirements or restrictions for co‐publication. Review authors accept responsibility for meeting any co‐publication requirements.

**I understand the commitment required to undertake a Campbell review, and agree to publish in the Campbell Library. Signed on behalf of the authors**:

**Form completed by: Vivian Welch**

**Date: Oct 25, 2017**

## Search strategy development

We developed a comprehensive search strategy with support from an information scientist (JM) for electronic databases and grey literature sources such as organizations active in deworming. The draft search strategy underwent review with PRESS (Peer Reviewed Electronic Search Strategies) (Sampson 2009) by John Eyers, information scientist of the Campbell International Development Group, and appropriate changes were made (Appendices A and B) to produce the finalised search strategy.

We identified relevant studies to inform the search strategy development: four randomised controlled trials and one cross‐sectional study that used propensity score matching (Azomahou, Diallo et al. 2012) that compare deworming combined with other interventions such as hygiene education (Taylor, Jinabhai et al. 2001), feeding (Azomahou, Diallo et al. 2012) or micronutrient supplementation (Biovin and Giordani 1993; Jinabhai, Taylor et al. 2001; Friis, Mwaniki et al. 2003) to placebo or active comparison groups.

## Electronic searches

The search included the following health and non‐health electronic databases: MEDLINE, CINAHL, LILACS, EMBASE, the Cochrane Library, Econlit, Internet Documents in Economics Access Service (IDEAS), Public Affairs Information Service (PAIS), Social Services Abstracts, Global Health CABI and CAB Abstracts. Grey literature databases were also included (e.g. thesis dissertations, System for Information on Grey Literature in Europe (SIGLE)‐ends in 2005).

We also searched websites of relevant organizations (UNICEF, Save the Children, Deworm the World, WHO, the World Bank, World Food Program, International Food Policy Research Institute (IFPRI) and Red Cross, Helen Keller International, Micronutrient Initiative, Global Alliance for Improved Nutrition (GAIN), Schools & Health: Health Nutrition, HIV and AIDS).

Other sources of reports and non‐published material were searched:

- AFROLIB Database (http://afrolib.afro.who.int/cgi‐bin/wxis.exe/iah/?IsisScript=iah/iah.xic&lang=I&base=afrolib)
- 3ie Database of Impact Evaluations (http://www.3ieimpact.org/database_of_impact_evaluations.html)
- BLDS British Library for Development Studies (http://blds.ids.ac.uk/)
- ELDIS (http://www.eldis.org/)
- International Clinical Trials Registry Platform ‐ Search Portal: http://www.who.int/trialsearch/
- East View Information Service Online Databases (httfp://online.eastview.com/index.jsp) – China, Russia and Soviet Union
- Index Medicus for the Western Pacific (WPRIM) (http://wprim.wpro.who.int/SearchBasic.php)
- South African Medical Database (SAMED) (http://www.mrc.ac.za/SamedSearch/

## Search strategy translations to different databases

**Database: Ovid MEDLINE(R) In‐Process & Other Non‐Indexed Citations and Ovid MEDLINE(R) <1946 to March 1, 2018**

**Search Strategy:**

————————————————————————–

C1 ‐ Database: Ovid MEDLINE(R) Epub Ahead of Print, In‐Process & Other Non‐Indexed Citations, Ovid MEDLINE(R) Daily and Ovid MEDLINE(R) <1946 to Present

Search Strategy:

——————————————————————————–

1 flukes.tw. (2019)

2 platyhelminth*.tw. (1257)

3 whipworm*.tw. (374)

4 whip worm*.tw. (10)

5 hookworm*.tw. (3929)

6 hookworm*.tw. (3929)

7 hook worm*.tw. (70)

8 roundworm*.tw. (1022)

9 round worm*.tw. (173)

10 geohelminth*.tw. (351)

11 ancylostoma*.tw. (1794)

12 Necator*.tw. (1224)

13 Ascaris.tw. (7058)

14 Ascaridida.tw. (114)

15 Ancylostoma.tw. (1694)

16 Necator americanus.tw. (673)

17 Trichuris.tw. (3235)

18 Trichuroidea.tw. (18)

19 Adenophorea.tw. (13)

20 Enoplida.tw. (36)

21 Ascaridida.tw. (114)

22 Platyhelminth*.tw. (1257)

23 Rotifera.tw. (262)

24 trichuriasis.tw. (412)

25 ascariasis.tw. (2070)

26 ancylostomiasis.tw. (480)

27 ascarid*.tw. (1811)

28 schistosomiasis.tw. (14919)

29 Schistosoma*.tw. (18130)

30 bilharziosis.tw. (187)

31 bilharzia*.tw. (2546)

32 exp Schistosoma/ (16316)

33 or/1‐32 (49421)

34 Albendazole/ (3970)

35 Mebendazole/ (1837)

36 exp Piperazines/ (69368)

37 Levamisole/ (4206)

38 exp Pyrantel/ (578)

39 Ivermectin/ (5621)

40 exp Anthelmintics/ (55618)

41 Ivermectin.tw. (5134)

42 Albendazole.tw. (4348)

43 Mebendazole.tw. (1757)

44 Piperazine*.tw. (7070)

45 Levamisole.tw. (4375)

46 pyrantel.tw. (692)

47 tiabendazole.tw. (21)

48 anthelmint*.tw. (8270)

49 *Antiplatyhelmintic Agents/ (232)

50 Anticestodal.tw. (27)

51 Antiplatyhelmintic.tw. (1)

52 Anti‐platyhelmintic.tw. (0)

53 Albendazole.tw. (4348)

54 Dichlorophen.tw. (55)

55 Niclosamide.tw. (561)

56 Bithionol.tw. (231)

57 Diamfenetide.tw. (4)

58 Nitroxinil.tw. (8)

59 Oxyclozanide.tw. (84)

60 Rafoxanide.tw. (130)

61 Schistosomicide*.tw. (126)

62 Antimony Potassium Tartrate.tw. (41)

63 Antimony Sodium Gluconate.tw. (2)

64 Hycanthone.tw. (322)

65 Lucanthone.tw. (112)

66 Niridazole.tw. (348)

67 Oxamniquine.tw. (390)

68 Praziquantel/ (3774)

69 Trichlorfon/ (1054)

70 metrifonate.tw. (341)

71 Artemisinins/ (5777)

72 (artesunate or artemether).tw. (3641)

73 or/34‐72 (141099)

74 (deworm* or de‐worm*).tw. (1231)

75 exp Anthelmintics/ or Anthelmintic*.tw. (57921)

76 74 or 75 (58628)

77 adolescent/ or exp child/ or exp infant/ (3260368)

78 child*.tw. (1220212)

79 paediatric*.tw. (53376)

80 pediatric*.tw. (241536)

81 youth.tw. (52104)

82 infant*.tw. (361795)

83 adolescen*.tw. (233224)

84 school age*.tw. (18911)

85 preschool.tw. (20982)

86 pre‐school.tw. (4113)

87 teen*.tw. (26911)

88 schoolchild*.tw. (12489)

89 or/77‐88 (3688849)

90 33 and 73 (7853)

91 76 or 90 (59419)

92 89 and 91 (7740)

93 exp animals/ not humans.sh. (4430459)

94 92 not 93 (7614)

**Database: Embase Classic+Embase <1947 to March 1, 2018**

**Search Strategy:**

————————————————————————–

1 whipworm*.tw. 500

2 whip worm*.tw. 20

3 hookworm*.tw. 4965

4 hookworm*.tw. 4965

5 hook worm*.tw. 124

6 roundworm*.tw. 1227

7 round worm*.tw. 244

8 pinworm*.tw. 722

9 pin worm*.tw. 41

10 flukes.tw. 2223

11 geohelminth*.tw. 428

12 ancylostoma.tw. 2008

13 Necator*.tw. 1527

14 Ascaris.tw. 9101

15 Ascaridida.tw. 114

16 Ancylostoma.tw. 2008

17 Necator americanus.tw. 917

18 Enterobius.tw. 1470

19 Oxyuroidea.tw. 46

20 Oxyurida.tw. 54

21 Trichuris.tw. 4036

22 Trichuroidea.tw. 20

23 Capillaria.tw. 880

24 Trichinella.tw. 4528

25 Strongyloid*.tw. 5806

26 Oesophagostomum.tw. 900

27 Oesophagostomiasis.tw. 38

28 Acanthocephala.tw. 739

29 Adenophorea.tw. 10

30 Enoplida.tw. 27

31 Secernentea.tw. 20

32 Ascaridida.tw. 114

33 Rhabditida.tw. 226

34 Cestoda.tw. 1639

35 Trematod*.tw. 6931

36 Turbellaria.tw. 218

37 Platyhelminth*.tw. 1274

38 Rotifera.tw. 267

39 trichuriasis.tw. 563

40 ascariasis.tw. 2570

41 trichinellosis.tw. 1417

42 Trichostrongyloidiasis.tw. 7

43 ancylostomiasis.tw. 375

44 enterobiasis.tw. 615

45 cestode*.tw. 4062

46 trematode*.tw. 5285

47 ascarid*.tw. 2299

48 schistosomiasis.tw. 17926

49 Schistosoma*.tw. 20916

50 or/1‐49 75524

51 Albendazole/ 12734

52 Mebendazole/ 5703

53 exp Piperazines/ 407195

54 Levamisole/ 11478

55 exp Pyrantel/ 686

56 Ivermectin/ 10378

57 exp Anthelmintics/ 123491

58 Ivermectin.tw. 6083

59 Albendazole.tw. 5849

60 Mebendazole.tw. 2164

61 Piperazine*.tw. 8663

62 Levamisole.tw. 5349

63 pyrantel.tw. 793

64 tiabendazole.tw. 84

65 anthelmint*.tw. 10084

66 praziquantel.tw. 4986

67 metrifonate.tw. 479

68 trichlorfon.tw. 449

69 (artesunate or artemether).tw. 5137

70 *Antiplatyhelmintic Agents/ 64

71 Anticestodal.tw. 30

72 Antiplatyhelmintic.tw. 1

73 Anti‐platyhelmintic.tw. 0

74 Albendazole.tw. 5849

75 Dichlorophen.tw. 67

76 Niclosamide.tw. 711

77 Bithionol.tw. 292

78 Diamfenetide.tw. 4

79 Nitroxinil.tw. 10

80 Oxyclozanide.tw. 93

81 Rafoxanide.tw. 140

82 Schistosomicide*.tw. 137

83 Antimony Potassium Tartrate.tw. 56

84 Antimony Sodium Gluconate.tw. 2

85 Hycanthone.tw. 434

86 Lucanthone.tw. 207

87 Niridazole.tw. 471

88 Oxamniquine.tw. 447

89 or/51‐88 528186

90 (deworm* or de‐worm*).tw. 1507

91 anthelmint*.tw. 10084

92 anthelmintic/ 11541

93 or/90‐92 17954

94 adolescent/ or exp child/ or exp infant/ 3410930

95 child*.tw. 1659449

96 paediatric*.tw. 91455

97 pediatric*.tw. 366079

98 youth.tw. 63904

99 infant*.tw. 473628

100 adolescen*.tw. 307333

101 school age*.tw. 25454

102 preschool.tw. 25586

103 pre‐school.tw. 6086

104 teen*.tw. 36196

105 schoolchild*.tw. 16384

106 or/95‐105 2349846

107 50 and 89 14863

108 107 or 93 28638

109 108 and 106 3370

110 limit 109 to dd=20150408‐20180301 431

111 whipworm*.tw. 500

112 whip worm*.tw. 20

113 hookworm*.tw. 4965

114 hookworm*.tw. 4965

115 hook worm*.tw. 124

116 roundworm*.tw. 1227

117 round worm*.tw. 244

118 pinworm*.tw. 722

119 pin worm*.tw. 41

120 flukes.tw. 2223

121 geohelminth*.tw. 428

122 ancylostoma.tw. 2008

123 Necator*.tw. 1527

124 Ascaris.tw. 9101

125 Ascaridida.tw. 114

126 Ancylostoma.tw. 2008

127 Necator americanus.tw. 917

128 Enterobius.tw. 1470

129 Oxyuroidea.tw. 46

130 Oxyurida.tw. 54

131 Trichuris.tw. 4036

132 Trichuroidea.tw. 20

133 Capillaria.tw. 880

134 Trichinella.tw. 4528

135 Strongyloid*.tw. 5806

136 Oesophagostomum.tw. 900

137 Oesophagostomiasis.tw. 38

138 Acanthocephala.tw. 739

139 Adenophorea.tw. 10

140 Enoplida.tw. 27

141 Secernentea.tw. 20

142 Ascaridida.tw. 114

143 Rhabditida.tw. 226

144 Cestoda.tw. 1639

145 Trematod*.tw. 6931

146 Turbellaria.tw. 218

147 Platyhelminth*.tw. 1274

148 Rotifera.tw. 267

149 trichuriasis.tw. 563

150 ascariasis.tw. 2570

151 trichinellosis.tw. 1417

152 Trichostrongyloidiasis.tw. 7

153 ancylostomiasis.tw. 375

154 enterobiasis.tw. 615

155 cestode*.tw. 4062

156 trematode*.tw. 5285

157 ascarid*.tw. 2299

158 schistosomiasis.tw. 17926

159 Schistosoma*.tw. 20916

160 or/111‐159 75524

161 Albendazole/ 12734

162 Mebendazole/ 5703

163 exp Piperazines/ 407195

164 Levamisole/ 11478

165 exp Pyrantel/ 686

166 Ivermectin/ 10378

167 exp Anthelmintics/ 123491

168 Ivermectin.tw. 6083

169 Albendazole.tw. 5849

170 Mebendazole.tw. 2164

171 Piperazine*.tw. 8663

172 Levamisole.tw. 5349

173 pyrantel.tw. 793

174 tiabendazole.tw. 84

175 anthelmint*.tw. 10084

176 *Antiplatyhelmintic Agents/ 64

177 Anticestodal.tw. 30

178 Antiplatyhelmintic.tw. 1

179 Anti‐platyhelmintic.tw. 0

180 Albendazole.tw. 5849

181 Dichlorophen.tw. 67

182 Niclosamide.tw. 711

183 Bithionol.tw. 292

184 Diamfenetide.tw. 4

185 Nitroxinil.tw. 10

186 Oxyclozanide.tw. 93

187 Rafoxanide.tw. 140

188 Schistosomicide*.tw. 137

189 Antimony Potassium Tartrate.tw. 56

190 Antimony Sodium Gluconate.tw. 2

191 Hycanthone.tw. 434

192 Lucanthone.tw. 207

193 Niridazole.tw. 471

194 Oxamniquine.tw. 447

195 or/161‐194 526649

196 (deworm* or de‐worm*).tw. 1507

197 anthelmint*.tw. 10084

198 anthelmintic/ 11541

199 or/196‐198 17954

200 adolescent/ or exp child/ or exp infant/ 3410930

201 child*.tw. 1659449

202 paediatric*.tw. 91455

203 pediatric*.tw. 366079

204 youth.tw. 63904

205 infant*.tw. 473628

206 adolescen*.tw. 307333

207 school age*.tw. 25454

208 preschool.tw. 25586

209 pre‐school.tw. 6086

210 teen*.tw. 36196

211 schoolchild*.tw. 16384

212 or/201‐211 2349846

213 160 and 195 14588

214 213 or 199 28364

215 214 and 212 3318

**Cochrane Library – CDSR, DARE, CENTRAL, EED, HTA**

**Search Name: deworming v2 March 1, 2018**

**Description:**

Search Name: deworming v2

Description:

ID Search

#1 helmint*:ti,ab,kw (Word variations have been searched)

#2 Ancylostoma duodenale

#3 Necator americanus

#4 Ascaris

#5 Enterobius vermicularis

#6 trichuris

#7 Strongyloid*

#8 hookworm*

#9 roundworm*

#10 pinworm*

#11 whipworm*

#12 schistosomiasis

#13 Schistosoma

#14 #1 or #2 or #3 or #4 or #5 or #6 or #7 or #8 or #9 or #10 or #11 or #13

#15 albendazole

#16 mebendazole

#17 piperazine

#18 levamisole

#19 pyrantel

#20 tiabendazole

#21 deworm*:ti,ab or de‐worm*:ti,ab

#22 #15 or #16 or #17 or #18 or #19 or #20 or #21

#23 #21 or #22

#24 #23 and #14

#25 deworm

#26 de‐worm

#27 deworming

#28 de‐worming

#29 anthelmint*

#30 anthelmintic

#31 #25 or #26 or #27 or #28 or #29 or #30

#32 #24 or #31

**CINAHL – Ebscohost March 1, 2018**

**Table:**

S24	S23 or S21
S23	S12 and S22
S22	S20 or S21
S21	“deworm*” or “de‐worm” or “anthelmint*” or “anthelmintic”
S20	S13 OR S14 OR S15 OR S16 OR S17 OR S18 OR S19
S19	(MH “Anthelmintics”)
S18	“pyrantel”
S17	“levamisole”
S16	(MH “Ranolazine”) OR “piperazines”
S15	“mebendazole”
S14	“albendazole”
S13	(MH “Anthelmintics+”) OR (MH “Antiprotozoal Agents+”)
S12	S1 OR S2 OR S3 OR S4 OR S5 OR S6 OR S7 OR S8 OR S9 OR S10 OR S11
S11	“bilharziosis”
S10	“bilharzia”
S9	(MH “Schistosomiasis”) OR “schistosomiasis”
S8	“pinworm” OR (MH “Enterobius”) OR (MH “Enterobiasis”)
S7	“necator”
S6	“roundworm”
S5	(MM “Hookworm Infections”)
S4	“trichuris trichiura”
S3	“whipworm”
S2	(MM “Helminths”)
S1	(MM “Trematodes”)

**WHO: database: LILACS, to March 1, 2018**

(tw:(deworm OR de‐worm OR anthelmintics OR anthelmintic)) AND (db:(“LILACS”)) AND (limit:(“humans”)) AND (instance:”ghl”)

**WHO (database: others) up to March 1, 2018**

(tw:(deworm OR de‐worm OR anthelmintics OR anthelmintic)) AND (db:(“WHOLIS” OR “WPRIM” OR “IMEMR” OR “IMSEAR” OR “AIM”)) AND (instance:”ghl”)

**ProQuest Social Services Abstracts, March 1, 2018**

**S1 or S2 or S3**

**S3** (deworm OR de‐worming OR de‐worm OR deworming OR anthelmintics OR anthelmintic) AND (child OR children OR school OR infant OR preschool OR teenager OR adolescent)

**S2** flukes or platyhelminth or whipworm or whip worm or hookworm or hook worm or roundworm or round worm or geohelminth or ancylostoma or necator or ascaris or Ascaridida or Ancylostoma or Trichuris or Trichuroidea or Adenophorea or Enoplida or Ascaridida or Platyhelminth or Rotifera or trichuriasis or ascariasis or ancylostomiasis or ascarid or schistosomiasis or Schistosoma or bilharziosis or bilharzias or schistosoma

**S1** Albendazole or Mebendazole or Piperazines or Levamisole or Pyrantel or Ivermectin or Anthelmintics or Ivermectin or Albendazole or Mebendazole or Piperazine or Levamisole or Pyrantel or Tiabendazole or anthelmint or Antiplatyhelmintic Agents or Anticestodal or Antiplatyhelmintic or Anti‐platyhelmintic or Albendazole or Dichlorophen or Niclosamide or Bithionol or Diamfenetide or Nitroxinil or Oxyclozanide or Rafoxanide or Schistosomicide or Antimony Potassium Tartrate or Antimony Sodium or Gluconate or Hycanthone or Lucanthone or Niridazole or Oxamniquine or Praziquantel or Trichlorfon or Metrifonate or Artemisinins or artesunate or artemether

**Proquest Econlit, up to March 1, 2018**

**S1 or S2 or S3**

**S3** (deworm OR de‐worming OR de‐worm OR deworming OR anthelmintics OR anthelmintic) AND (child OR children OR school OR infant OR preschool OR teenager OR adolescent)

**S2** flukes or platyhelminth or whipworm or whip worm or hookworm or hook worm or roundworm or round worm or geohelminth or ancylostoma or necator or ascaris or Ascaridida or Ancylostoma or Trichuris or Trichuroidea or Adenophorea or Enoplida or Ascaridida or Platyhelminth or Rotifera or trichuriasis or ascariasis or ancylostomiasis or ascarid or schistosomiasis or Schistosoma or bilharziosis or bilharzias or schistosoma

**S1** Albendazole or Mebendazole or Piperazines or Levamisole or Pyrantel or Ivermectin or Anthelmintics or Ivermectin or Albendazole or Mebendazole or Piperazine or Levamisole or Pyrantel or Tiabendazole or anthelmint or Antiplatyhelmintic Agents or Anticestodal or Antiplatyhelmintic or Anti‐platyhelmintic or Albendazole or Dichlorophen or Niclosamide or Bithionol or Diamfenetide or Nitroxinil or Oxyclozanide or Rafoxanide or Schistosomicide or Antimony Potassium Tartrate or Antimony Sodium or Gluconate or Hycanthone or Lucanthone or Niridazole or Oxamniquine or Praziquantel or Trichlorfon or Metrifonate or Artemisinins or artesunate or artemether

**Proquest Public Affairs Information Service (PAIS), up to March 1, 2018**

**S1 or S2 or S3**

**S3** (deworm OR de‐worming OR de‐worm OR deworming OR anthelmintics OR anthelmintic) AND (child OR children OR school OR infant OR preschool OR teenager OR adolescent)

**S2** flukes or platyhelminth or whipworm or whip worm or hookworm or hook worm or roundworm or round worm or geohelminth or ancylostoma or necator or ascaris or Ascaridida or Ancylostoma or Trichuris or Trichuroidea or Adenophorea or Enoplida or Ascaridida or Platyhelminth or Rotifera or trichuriasis or ascariasis or ancylostomiasis or ascarid or schistosomiasis or Schistosoma or bilharziosis or bilharzias or schistosoma

**S1** Albendazole or Mebendazole or Piperazines or Levamisole or Pyrantel or Ivermectin or Anthelmintics or Ivermectin or Albendazole or Mebendazole or

Piperazine or Levamisole or Pyrantel or Tiabendazole or anthelmint or Antiplatyhelmintic Agents or Anticestodal or Antiplatyhelmintic or Anti‐platyhelmintic or Albendazole or Dichlorophen or Niclosamide or Bithionol or Diamfenetide or Nitroxinil or Oxyclozanide or Rafoxanide or Schistosomicide or Antimony Potassium Tartrate or Antimony Sodium or Gluconate or Hycanthone or Lucanthone or Niridazole or Oxamniquine or Praziquantel or Trichlorfon or Metrifonate or Artemisinins or artesunate or artemether

**CAB Abstracts and GLOBAL HEALTH CAB INTERNATIONAL, searched using CAB direct interface up to April 12, 2018**

((random* OR RCT* OR trial* OR placebo OR (double AND blind*))) AND ((deworm* OR de‐worming OR de‐worm* OR deworming OR anthelmintics OR anthelmintic OR anthelminth* OR antihelmint*)) AND ((child OR children OR school* OR infant* OR preschool OR teen* OR adolescen*))
